# Changes in Corneal Epithelial Thickness Induced by Topical Antiglaucoma Medications

**DOI:** 10.3390/jcm10163464

**Published:** 2021-08-05

**Authors:** Myungsik Nam, Sun Woong Kim

**Affiliations:** Department of Ophthalmology, Yonsei University Wonju College of Medicine, 20 Ilsan-ro, Wonju 26426, Gangwon-do, Korea; nms908@naver.com

**Keywords:** corneal epithelial thickness, glaucoma, topical glaucoma medication, Fourier domain optical coherence tomography, benzalkonium chloride

## Abstract

Corneal thickness measurement is important for assessing intraocular pressure in patients with glaucoma. This study investigated the changes in corneal epithelial thickness (CET) induced by antiglaucoma medications and explored the factors affecting CET measurement. CET was measured over a 9.0 mm diameter area by using Fourier domain optical coherence tomography in 125 patients with primary open-angle glaucoma and 125 age-matched controls without glaucoma. The influence of sex, age, benzalkonium chloride (BAK)-containing instillations, disease severity, and types and numbers of medications was analyzed using simple and multiple regression analyses. CET over 25 sectors was smaller in the glaucoma group than in the control group (mean difference of 4.2 µm in the central 2.0 mm zone; 52.8 ± 3.6 vs. 48.5 ± 3.9, *p* < 0.001). Simple regression analysis revealed age, use of β-blockers, prostaglandin, carbonic anhydrase inhibitors, total number of medications, and number of daily BAK-containing instillations were associated with a thinner epithelium. Multiple regression analysis revealed β-blockers, prostaglandin, and number of BAK-containing instillations were significant factors. Use of β-blockers and number of BAK-containing instillations were also associated with a thinner epithelium in the monotherapy subgroup analysis. CET was significantly smaller in patients with glaucoma receiving topical medications and was affected by the use of β-blockers, prostaglandin, and BAK.

## 1. Introduction

Measurement of corneal thickness has played an important diagnostic role in the assessment of intraocular pressure (IOP) in patients with glaucoma [1,2,3]. Central corneal thickness (CCT) is also an independent risk factor for glaucoma, and potential factors affecting corneal thickness measurements or their association with IOP measurements have been extensively studied [3,4]. The recent development of topographic mapping using Fourier domain optical coherence tomography (FD-OCT) has enabled the measurement of corneal epithelial thickness (CET) as well as corneal thickness in the clinical routine with various clinical applications [5,6,7]. The corneal epithelium is one of the main structures that undergo degenerative alterations in ocular diseases. Hence, CET measurement has been suggested as an objective indicator for monitoring ocular surface health [8]. Previous studies have reported alterations in CET in patients with dry eye syndrome [9,10,11], and it plays a role in the early detection of keratoconus [12,13].

Ocular surface discomforts are more common in patients with glaucoma than in those without glaucoma. Since patients with glaucoma often require the long-term use of multiple eyedrops, the possible adverse effects of these drugs on the ocular surface have been important topics of research [14,15,16]. Previous studies that investigated the effects of glaucoma eyedrops on CET reported that patients with glaucoma had uniformly reduced CETs compared to normal controls [17,18,19]. Most studies, however, failed to detect any association between CET and treatment parameters. Many studies showed that benzalkonium chloride (BAK), a common preservative used in ophthalmic eyedrops, induced ocular surface discomfort and epithelial toxicity [20,21,22]. The adverse effects of BAK on the corneal epithelium have been reported in various studies, but its effect on altering CET has not yet been demonstrated.

Therefore, we aimed to investigate the influence of sex, age, BAK-containing instillations, disease severity, and types and numbers of topical medications on CET and stromal thickness in patients with glaucoma.

## 2. Materials and Methods

### 2.1. Participants

This retrospective cohort study included 125 patients with primary open-angle glaucoma and 125 age-matched controls without glaucoma who were evaluated between March 2019 and March 2021. A complete ophthalmologic examination, including slit-lamp examination, IOP measurement, corneal thickness measurement, visual field analysis, OCT, and fundus examination, was performed, and patients with any corneal pathologies, such as corneal opacity, corneal dystrophies, keratoconus, a history of contact lens wear, and prior ocular surgery within 6 months, were excluded. Patients with glaucoma treated using the same medication for at least 6 months were included in the analysis. The control group comprised individuals presenting for regular check-ups. Individuals using any topical eyedrops except antiglaucoma medications were excluded from both groups. The CET and total thickness data were obtained using the RTVue (Software Version: 2018, 1, 0, 43) FD-OCT system (Optovue Inc., Fremont, CA, USA) with a corneal adaptor module working at 830 nm wavelength. CET maps were generated by an automatic algorithm and divided into a total of 25 sectors over a 9 mm corneal diameter: a central 2 mm zone, eight paracentral zones within an annulus between the 2 and 5 mm diameter rings, eight mid-peripheral zones within an annulus between the 5 and 7 mm diameter rings, and eight peripheral zones within an annulus between the 7 and 9 mm diameter rings. The CET standard deviation within the central 7 mm area generated by the software was recorded as thickness variability. Stromal thickness was calculated by subtracting the epithelial thickness from the corneal thickness in the corresponding area. All ophthalmic measurement data obtained from only one randomly selected eye of each individual were included for statistical analysis. The study protocol was approved by the review board of our institute (CR321015), and the study was conducted according to the tenets of the Declaration of Helsinki. The need for informed consent was waived because of the retrospective nature of the study.

### 2.2. Statistical Analysis

Statistical analyses were performed using IBM SPSS Statistics for Windows, Version 25.0 (IBM Corp., Armonk, NY, USA). Student’s t-test was performed to compare continuous variables between patients with glaucoma and the age-matched controls. Simple linear regression was used to determine the association of age, sex, number of daily BAK-containing instillations, and types and numbers of medications with CET and stromal thickness. Multiple linear regression analyses were then conducted to investigate the influences of the associated factors derived from the simple regression analysis on CET and stromal thickness. A subgroup of 56 individuals using only one eyedrop was further analyzed to validate the effect of individual medication and BAK-containing instillations. Fixed combination medication users were included in the monotherapy subgroup. Values with *p* < 0.05 were considered statistically significant.

## 3. Results

### 3.1. CET Profiles

**Figure 1:**
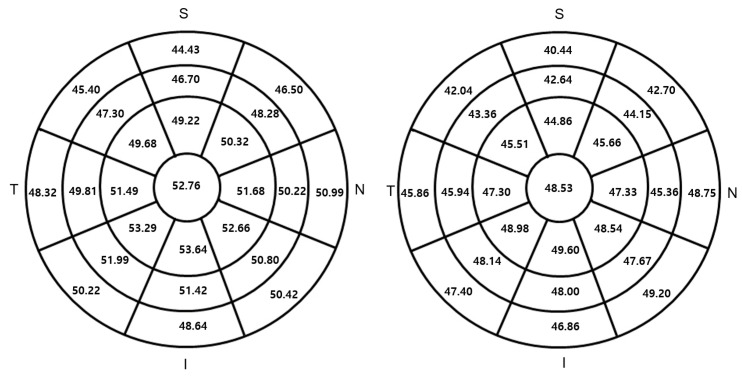
Topographic corneal epithelial thickness maps of the control (**left**) and glaucoma (**right**) groups.

This study analyzed data from 125 patients with glaucoma with a mean age of 66.7 ± 11.0 years and 125 controls with a mean age of 65.1 ± 8.9 years (Table 1). No differences were observed in age or sex distribution between the control and glaucoma groups. In our 250-individual cohort comparison, CET in all 25 sectors over the 9.0 mm diameter area was smaller in the glaucoma group than in the control group (Figure 1). However, CCT, central stromal thickness, and epithelial thickness variabilities were not different in the two groups. In both groups, superior epithelial thickness was smaller than inferior epithelial thickness, and epithelial thickness variability was greater in females than in males. The mean difference in epithelial thickness between the glaucoma and control groups was similar in the superior and inferior cornea (Figure 1).

### 3.2. Association between Glaucoma Medication and Corneal Thickness

Simple regression analysis indicated that age, visual field index, use of β-blockers, prostaglandin, carbonic anhydrase inhibitors (CAIs), α-agonists, total number of medications, and number of daily BAK-containing instillations were associated with a thinner epithelium. Age was inversely proportional to corneal stromal and epithelial thicknesses in the glaucoma group, but it was not associated with CET in the control group (Table 2). Multiple regression analysis indicated that the use of β-blockers, prostaglandin, and number of BAK-containing instillations remained significant factors even after controlling for the other factors. The total number of medications was excluded from the multiple regression analysis owing to collinearity (Table 3).

### 3.3. Association between Glaucoma Medication and CET in the Monotherapy Medication Group

To demonstrate the effect of individual eyedrops without confounding factors, 56 eyes that were receiving only one eyedrop were further analyzed. This analysis revealed no differences in CET, stromal thickness, CCT, and epithelial thickness variability between individuals divided by the presence of preservatives in the administered eyedrop (Table 4). The use of β-blockers showed a significant difference in epithelial thickness (51.1 vs. 49.1, *p* = 0.029), whereas the use of prostaglandin, CAIs, α-agonists, and BAK did not induce significant differences (Table 5). However, a linear association was observed between epithelial thickness and the use of β-blockers (R = 0.292, *p* = 0.029) and number of BAK-containing instillations (R = 0.288, *p* = 0.031; Table 6).

## 4. Discussion

The anterior segment OCT imaging has attracted increasing attention because it is capable of indicating ultrastructural or functional changes of the ocular surface, which is useful for detecting corneal sublayer changes in various conditions such as keratoconus, laser refractive surgery, corneal crosslinking, orthokeratology, corneal dystrophies, and chemical burns [12,23,24,25,26,27,28,29]. Corneal epithelial imaging via FD-OCT has recently become available and serves as a practical tool for in vivo epithelial mapping. Specifically, damage to the ocular surface can be evaluated using OCT epithelial thickness mapping because of its high repeatability and noninvasive nature. In this context, recent studies suggested topographic epithelial mapping as an objective measurement to assess patients with dry eye [8,9,10,11]. Cui et al. demonstrated the presence of greater epithelial thickness variability and a thinner epithelium in the superior sector of patients with severe dry eye [10]. Abou Shousha et al. reported higher thickness variability in dry eye and showed that thickness variability correlated with a patient’s symptoms [11]. They also showed a significant reduction in epithelial thickness variability after treatment and suggested that epithelial thickness profiles could be used during follow-up to monitor a patient’s response to treatment. Kanellopoulos and Asimellis also reported epithelial thickening and increased thickness variability in patients with dry eye and suggested a diagnostic role of OCT-based epithelial thickness measurement in such patients [8,9].

Given the increased prevalence of dry eye or ocular surface discomfort in patients with glaucoma, previous studies investigated the effects of IOP-lowering eyedrops on CET and reported smaller CETs in patients with glaucoma [17,18,19]. Since commercially available antiglaucoma eyedrops often contain preservatives, many concerns have been raised regarding the effect of these preservatives on ocular surface health. Specifically, the commonly used preservative BAK has been known to cause several detrimental effects on the corneal and conjunctival epithelium [15,20,21,22]. As such, the observed changes in CET may be attributed to BAK toxicity, to the direct effects of active ingredients in antiglaucoma eyedrops, or less likely to the intrinsic pathologic mechanism of glaucoma.

This study examined CET and stromal thickness in patients with glaucoma and aimed to detect factors that could affect the thickness profiles. When compared to an age-matched control group without glaucoma, the glaucoma group showed statistically significant reduction in CET in all 25 sectors over a 9.0 mm diameter area. Previous studies have reported reduced CETs in patients with glaucoma, but only few studies revealed significant factors affecting the reduction in CET [17,18,19]. Many studies used manual measurements of the central cornea and examined a relatively small number of subjects. Doğan et al. examined 153 glaucomatous eyes using manual caliper-based measurements and reported reduced CETs in patients with glaucoma. However, they found no significant difference in central CET with regard to the glaucoma type, duration of therapy, number of drugs used, and number of daily drug instillations [17]. Batawi et al. also performed caliper-based measurements of central CET on OCT images of 58 male patients with glaucoma and reported reduced central CETs and corneal stromal thicknesses in these patients. They also reported that the number of topical medications used correlated significantly with CET [18]. Halkiadakis et al. compared 62 patients with glaucoma to an identical number of controls and demonstrated that the patients with glaucoma had uniformly reduced corneal thicknesses. They also identified that the use of β-blockers was associated with a thinner epithelium by using the same device used in the present study [19]. However, they did not find any association between CET and the number of medications, number of instillations, and years of treatment. In contrast, the present study found that both the total number of medications and severity of glaucoma were associated with reduced epithelial thicknesses. Although severity of glaucoma was correlated with CET in our simple regression analysis, we believe that this association was caused by a linear relationship between increased medication and severity rather than a direct association between CET and severity. Differences in the number of medications, preservatives, disease severity, race or ethnicity, and duration of therapy are factors that could have contributed to these different results.

Surprisingly, no previous studies have evaluated the effect of the preservative BAK on epithelial thickness, and most studies have not reported the differential results based on the presence of BAK in the administered eyedrops. The current study therefore focused on the effects of individual medications and BAK on CET and stromal thickness. We identified that both the total number of medications and the severity of glaucoma were significant confounding factors because they correlated with many variables and were associated with reduced epithelial thicknesses. Our analysis of the monotherapy subgroup has therefore provided insights on the effects of individual medications and BAK. Our data demonstrated that β-blockers reduced CET and the number of BAK-containing instillations was significantly associated with epithelial thinning. An association between β-blockers and CET has also been observed in previous studies [18,19]. Interestingly, β-blockers have been reported to be effective in restoring myopic regression by reducing CET in patients with epithelial hyperplasia following myopic laser refractive surgery [30,31]. This indicates that β-blockers may induce a reduction in epithelial thickness, but the underlying mechanism needs further clarification. Another explanation could be made by the potential effect on tear production. As CET measurement by OCT includes the precorneal tear film, alteration on the tear layer may potentially affect CET measurement. Systemic β-blockers have been reported to reduce tear production, and previous studies supported that topically applied β-blockers may act systemically [32,33,34]. Recently, a previously poorly understood link between the cardiovascular system (heart rate) and the lacrimal functional unit (tear film dynamics) was revealed by Napoli et al. [35]. This association supports a hypothesis that β-blockers may affect the measurement of CET by alteration of tear film thickness. Moreover, the effect of BAK on CET also could be explained by alteration of tear film thickness as it has been known to cause tear film instability.

Notably, the presence of preservatives in monotherapy drugs did not induce any significant alterations in epithelial thickness (Table 4), but it was a significant factor in cases of multiple drug use. Furthermore, the use of BAK seemed to induce more prominent alterations in CET even though the difference was not statistically significant (Table 5 and Table 6). Given the fact that most patients with glaucoma were treated using more than one medication, our finding suggests the augmented effects or dose-dependent effects of BAK on the corneal epithelium. The use of β-blockers and number of BAK-containing instillations were significant factors that induced a reduction in epithelial thickness after controlling for other confounding factors in our multiple regression analysis. The use of prostaglandin also induced a reduction in CET, but it remains unclear whether this was induced by the active ingredient prostaglandin. As reduced CET was not observed in the monotherapy subgroup analysis, this observation might be attributed to a combination of factors. Notably, previous studies have shown that topical prostaglandin significantly reduced CCT with some conflicting results [36,37,38]. The current study also found that prostaglandin had an effect on reducing CCT, but its effects on epithelial thickness warrant further research.

We also investigated the topographic epithelial thickness maps of the glaucoma and control groups. Overall, the topographic profiles were similar in the two groups, and previous studies reported that the superior and temporal epithelial thicknesses were smaller than the inferior and nasal epithelial thicknesses, respectively [6,39]. An interesting finding of our study was the absence of a significant difference in thickness variability between the glaucoma and control groups. This indicated that the reduction in epithelial thickness occurred in the entire studied area and was induced by medication and was not due to the detrimental effects of dry eye. We also did not find any significant difference in central stromal thickness or CCT between the glaucoma and control groups, but some studies reported reduced CCTs in patients with glaucoma as well. These different results may be attributed to differences in the patient populations and measurement techniques. Our data also indicated that topical antiglaucoma medications, except for prostaglandin, induced alterations mostly in CET.

This study has several limitations. First, this study did not include the duration of treatment as a variable. Since our hospital is a tertiary care center, only a limited number of individuals are newly diagnosed, and it is difficult to know exactly how long the patients have been on medications. However, previous studies have reported that there was no correlation between CET and glaucoma treatment duration [17,19]. Second, we could not clarify the effects of BAK. Our monotherapy subgroup data suggested that the use of BAK-containing eyedrops did not induce alterations in epithelial thickness, even though it appeared to be an important factor in 125 individuals. Notably, the difference in CET was more prominent in the comparison between BAK-containing eyedrops and BAK-free eyedrops rather than when including all preservative-containing eyedrops in one group. Although many previous studies have reported BAK toxicity, few studies have reported the detrimental effects of other preservatives such as Purite or Polyquad. This finding also warrants further validation. The total number of medications and disease severity were significantly correlated with epithelial thickness, and these could be potential confounding factors in our analysis. The implication of thinner CET on the severity of glaucoma needs to be further investigated in the future. Third, the CET data of current OCT include precorneal tear film, the thickness of which has been reported to be highly variable. Recent studies indicated that the anterior surface of the corneal epithelium shows a roughness of approximately 5 µm, which may mirror the wavy motion of the tear film during visual fixation [40,41]. This is an apparent limitation of OCT measurement and a source of possible uncertainty in our results. Since RTVue software showed an average thickness value over a defined area, the observed findings may reflect combined effects of alteration of precorneal tear film thickness and CET. More advanced imaging technology may be needed to differentiate effects on tear film and epithelial thickness in detail. Nevertheless, despite these limitations, our results are in good agreement with those observed in real-world practice, wherein patients receiving monotherapy do not experience severe ocular surface discomforts and those receiving multiple drugs usually experience ocular surface discomforts more frequently. Finally, we could not demonstrate any increase in epithelial thickness after switching medications from the ones containing preservatives to those that are preservative-free in our longitudinal follow-up data (data not shown). This requires further investigation in a future prospective study. The effect of BAK could be clarified after switching medication from BAK-containing drops to preservative-free drops. The determination of when the alteration of CET occurs is another interesting topic. These issues require further investigations in future prospective studies.

## 5. Conclusions

The present study demonstrated that the CET profiles of patients with glaucoma receiving topical medications were uniformly reduced when compared to those of controls without glaucoma. The observed alterations were affected by the use of β-blockers, prostaglandin, and BAK, and the total number of medications and disease severity were confounding factors.

## Figures and Tables

**Table 1:** Comparison of corneal epithelial thickness between the control and glaucoma groups.

Patient Characteristics	Control (*n* = 125)	Glaucoma (*n* = 125)	*p* Value
Age (years)	65.1 ± 8.9	66.7 ± 11.0	0.191
Sex	62:63	68:57	0.527
CCT	525.0 ± 29.6	520.7 ± 31.7	0.269
CST	472.2 ± 29.5	472.3 ± 29.5	0.987
**Epithelium (µm)**			
Central	52.8 ± 3.6	48.5 ± 3.9	<0.001 *
Paracentral	51.5 ± 3.4	47.2 ± 4.0	<0.001 *
Mid-peripheral	49.6 ± 3.3	45.8 ± 3.8	<0.001 *
Peripheral	48.1 ± 3.4	45.4 ± 4.1	<0.001 *
Thickness variability	2.9 ± 1.0	3.0 ± 1.1	0.304

* Statistically significant difference at *p* < 0.05; Student’s *t*-tests. Values are presented as the mean ± standard deviation. CCT: central corneal thickness, CST: central stromal thickness.

**Table 2:** Factors affecting corneal epithelial and stromal thicknesses analyzed using a simple regression analysis.

	Simple Regression					
	B	R	*p*	B	R	*p*
Central Epithelium				Central Stroma		
Age	−0.068	0.189	0.035	−0.543	0.189	0.034
Sex	0.029	0.004	0.967	−0.400	0.006	0.944
Medication number	−1.874	0.402	<0.001	−3.682	0.099	0.271
BAK-containing instillations	−1.413	0.428	<0.001	−2.085	0.079	0.379
β-blockers	−3.257	0.390	<0.001	−4.118	0.062	0.493
PG	−2.147	0.238	0.007	−11.912	0.166	0.064
CAIs	−2.223	0.274	0.002	1.514	0.023	0.795
α-agonists	−1.397	0.176	0.049	−0.519	0.008	0.927
VFI	0.042	0.346	<0.001	−0.104	0.108	0.231

BAK: Benzalkonium chloride, PG: prostaglandin, CAIs: carbonic anhydrase inhibitors, VFI: visual field index.

**Table 3:** Factors affecting corneal epithelial and stromal thicknesses analyzed using a multiple regression analysis.

	Multiple Regression		
	B	R	*p*
Central epithelium		0.550	<0.001
Age	−0.016		0.597
BAK-containing instillations	−0.777		0.016
β-blockers	−2.449		0.038
PG	−1.690		0.024
CAIs	0.575		0.589
α-agonists	−0.209		0.756
VFI	0.017		0.117

**Table 4:** Effect of preservatives on corneal thickness in the monotherapy group.

Patient Characteristics	Preservative-Containing Medication (*n* = 37)	Preservative-Free Medication (*n* = 19)	*p* Value
Age (years)	65.9 ± 10.8	63.1 ± 11.6	0.376
CCT	529.1 ± 31.4	526.6 ± 29.6	0.775
CST	479.0 ± 31.1	475.9 ± 29.7	0.719
**Epithelium (µm)**			
Central	50.1 ± 3.3	50.7 ± 3.3	0.502
Paracentral	49.1 ± 3.6	49.6 ± 2.9	0.565
Mid-peripheral	47.4 ± 3.6	48.1 ± 2.7	0.522
Peripheral	46.7 ± 3.5	47.47 ± 3.7	0.499
Thickness variability	2.8 ± 1.1	2.8 ± 0.81	1.000

**Table 5:** Effect of glaucoma medication on corneal thickness in the monotherapy group.

Medication		*n*	CET	CST	CCT
β-blockers	Yes	23	49.1 ± 3.1	476.8 ± 28.3	526.0 ± 28.2
	No	33	51.1 ± 3.2	478.8 ± 32.2	529.8 ± 32.4
	*p*		0.029	0.818	0.646
PG	Yes	29	50.3 ± 3.1	471.0 ± 29.8	521.3 ± 29.6
	No	27	50.3 ± 3.5	485.4 ± 29.7	535.7 ± 30.3
	*p*		0.985	0.075	0.077
BAK	Yes	30	49.5 ± 3.0	472.4 ± 30.0	521.9 ± 29.7
	No	26	51.2 ± 3.4	484.4 ± 30.1	535.5 ± 30.5
	*p*		0.059	0.143	0.096

PG: prostaglandin.

**Table 6:** Factors affecting corneal epithelial thickness in the monotherapy group.

	Simple Regression					
	B	R	*p*	B	R	*p*
Central Epithelium				Central Stroma		
Age	−0.068	0.189	0.279	−1.220	0.443	0.001
BAK-containing instillations	−1.196	0.288	0.031	−4.855	0.126	0.353
β-blockers	−1.930	0.292	0.029	−1.931	0.032	0.818
PG	0.017	0.003	0.985	−14.444	0.240	0.075
CAIs	−0.638	0.090	0.590	6.893	0.105	0.440
α-agonists	1.005	0.113	0.405	20.695	0.252	0.061
VFI	0.032	0.180	0.188	0.142	0.083	0.548

## Data Availability

The data presented in this study are available on request from the corresponding author. The data are not publicly available due to ethical issue.
